# Evaluating the efficacy of machine learning in predicting postherpetic neuralgia: a systematic review and meta-analysis

**DOI:** 10.3389/fneur.2025.1632682

**Published:** 2025-09-15

**Authors:** Zheng Lin, Hongfei Wang, Chenxi Ma, Ruyi Ju, Yi Cao, Ping Lin

**Affiliations:** ^1^The First Affiliated Hospital of Zhejiang Chinese Medical University, Hangzhou, Zhejiang, China; ^2^Hangzhou Third People’s Hospital Affiliated to Zhejiang Chinese Medical University Excluding The First Affiliated Hospital of Zhejiang Chinese Medical University, Hangzhou, Zhejiang, China

**Keywords:** machine learning, postherpetic neuralgia, herpes zoster, prediction model, logistic regression

## Abstract

**Introduction:**

The prediction of postherpetic neuralgia (PHN) is of great clinical significance. PHN prediction based on machine learning have received extensive attention in recent years. This study aims to conduct a comprehensive evaluation of machine learning in PHN prediction and provide guidance for the future models.

**Method:**

The system retrieved the relevant literatures published in the PubMed, Web of Science, Embase and Cochrane Library databases from the establishment of the database to May 2025. Literature screening and data extraction were conducted in accordance with the PRISMA guidelines. According to the heterogeneity, the fixed-effect or the random-effect model was selected for data synthesis. The potential sources of heterogeneity were further explored through subgroup analysis, sensitivity tests and meta-regression. Funnel plots and Deeks’ tests were used to evaluate the possible publication biases.

**Result:**

The main meta-analysis included 41 models from 14 studies. The results showed that machine learning demonstrated excellent performance in predicting PHN (sensitivity: 0.81, 95% confidence interval (CI): 0.74–0.86; specificity: 0.84, 95% CI: 0.79–0.88; area under the curve: 0.90, 95% CI: 0.87–0.92). Meta-regression analysis indicates that the source of the data set, model selection, and the choice of predictors are the main reasons leading to heterogeneity. Subgroup analysis showed that the training set model outperformed the validation set model. Logistic regression and other machine learning had varying strengths and weaknesses. Serum data or omics analysis did not significantly enhance model performance.

**Conclusion:**

Machine learning represents a promising approach for the prediction of PHN. However, most of the existing models face issues like lack of external validation, overfitting, and insufficient reporting standardization. This has raised doubts about whether the current PHN prediction models can still maintain a high prediction accuracy when extended to external data. To improve future models, we recommend conducting strict external validation, clearly reporting cutoff values (balanced, positive, and negative), and adhering to international predictive model reporting standards. When applicable, ensemble learning and pain trajectory analyses should also be considered.

**Systematic review registration:**

This study was registered in the Prospective Register of Systematic Reviews (PROSPERO; CRD420251054364).

## 1 Introduction

Herpes zoster (HZ) is caused by the reactivation of the varicella-zoster virus (VZV), the lifetime prevalence rate of HZ worldwide is 25–50% (1). Postherpetic neuralgia (PHN) is the most common complication of HZ, usually defined as pain that persists for more than 90 days after the HZ rash heals (2). The incidence of PHN varies from 5 to 20% among different age groups (3). PHN has a significant impact on both the quality of life and the economy of patients (4).

The adjuvanted herpes zoster subunit vaccine has been proven to have good safety and efficacy and is suitable for the prevention of HZ and PHN (5). However, the actual vaccination rate situation is deeply worrying. A meta-analysis shows that less than half of the people indicated their willingness to get vaccinated against shingles. The main reasons for the low willingness to be vaccinated include: lack of trust in the effectiveness and safety of the shingles vaccine, economic burden issues, and lack of understanding of vaccine information (6). In this situation, a large number of people are at risk of PHN, which makes accurate prediction of PHN remain an important goal that needs to be achieved in clinical practice.

In recent years, the application of machine learning (ML) in the field of medicine has become increasingly widespread (7). In the field of predicting PHN, most ML prediction models take whether PHN occurs as the binary classification result and are constructed using the predictors collected by patients at their first visit. From the perspective of clinical application, compared with traditional statistical methods, the results output by ML have more practical application value because they can provide direct support for clinical decision-making. For instance, for patients whose ML predicts they may suffer from PHN, doctors can implement more effective pain intervention measures to reduce the probability of PHN occurrence, thereby achieving the goal of precise treatment.

A large number of studies have reported PHN ML prediction models constructed based on their own datasets. However, due to the high sensitivity of machine learning models to data, the current models may have problems such as overfitting and limited generalization ability. At present, there is a lack of systematic reviews on the effectiveness of ML in predicting PHN. Therefore, in this study, we used meta-analysis to conduct a comprehensive evaluation of the predictive value of ML for PHN. The aim is to evaluate the advantages and limitations of current ML models in predicting PHN, systematically summarize the types of models and predictors used, and provide research directions and improvement suggestions for the development of future models.

## 2 Methods

### 2.1 Research design

This study was conducted in accordance with the Preferred Reporting Item for Systematic Reviews and Meta-Analyses (PRISMA) guidelines (8). The PRISMA checklist can be seen from Supplementary Table 1. This study was registered in the Prospective Register of Systematic Reviews (PROSPERO; CRD420251054364).

### 2.2 Search strategy

Articles published between the establishment of the databases and May 2025 were retrieved in PubMed, Web of Science, Embase and the Cochrane Library databases. We constructed the retrieval strategy based on the PICO principle (population, intervention, control, and outcomes). Population: patients with HZ. Intervention: establish ML model for predicting PHN. Control: the gold standard for diagnosing PHN. Outcomes: The predicted result, including true positive (TP), false positive (FP), true negative (TN) and false negative (FN). The research on search strategies is summarized in Supplementary Table 2.

### 2.3 PICOS framework

The parameters of this systematic review, as defined by the PICOS framework, were as follows:

*Participants:* Patients clinically diagnosed with HZ or those with a disease code of HZ.
*Index:* Utilizing ML to analyze data of patients with HZ for the prediction of PHN.
*Comparator:* Prognostic factor (occurrence of PHN vs. non-occurrence of PHN after HZ).
*Outcome:* The accuracy of predicting the occurrence of PHN in HZ patients based on their clinical characteristics.
*Study design:* Studies with cohort, case–control, and cross-sectional designs.

### 2.4 Eligibility criteria

Inclusion criteria: 1. All the included literatures were published in English. 2. The included study adopted the clear definitions of HZ and PHN. The population was patients diagnosed with HZ, and the model endpoint was set as the onset of PHN. 3. Modeling was carried out using at least one ML method. 4. Reporting data that can infer TP, FP, TN and FN of the model.

Exclusion criteria: 1. Duplicate studies, non-English publications, and studies with missing or non-convertible data were excluded. 2. Studies that only reported the corresponding risk factors of PHN using logistic regression (LR) but did not model for prediction were excluded.

Titles and abstracts of potentially eligible studies were screened by two independent researchers (Zheng Lin and Hongfei Wang), and the disagreement were resolved by the third independent researcher (ChenXi Ma). Subsequently, the full text of these studies was systematically assessed to further confirm whether they met the inclusion criteria.

### 2.5 Data collection

For the publications included in the analysis, two independent researchers (Zheng Lin and Wang Hongfei) systematically collected the following information: 1. basic information about the study, including authors, publication year, country, study design, sample size, and diagnostic criteria for HZ and PHN. 2. Information related to the models, including the dataset, model type, and predictors. 3. The performance indicators of the model, including the area under the curve (AUC), sensitivity, specificity, TP, FP, TN and FN.

### 2.6 Assessment of risk of bias

The Prediction Model Risk of Bias Assessment Tool (PROBAST) is suitable for evaluating the risk of bias and applicability of the original studies for the development or validation of multivariate diagnostic/prognostic models (9). Two independent researchers (Zheng Lin and Wang Hongfei) used the PROBAST to evaluate each included study, and the disagreement were resolved by the third independent researcher (ChenXi Ma).

### 2.7 Statistical analysis

All analyses in this study were done based on Stata 14.0 (Stata Corporation, Texas, United States) and R 4.4.2 (R Foundation for Statistical Computing, Vienna, Austria).

The main statistical measures used in meta-analysis were sensitivity and specificity. Draw the Summary Receiver Operating Characteristic (SROC) curve summarized by the sensitivity and specificity of each study. The diagnostic value of the ML is reflected through the Fagan diagram and the distribution scatter diagram and evaluated by positive likelihood ratio (PLR) and negative likelihood ratio (NLR).

I^2 was used to evaluate the heterogeneity level of the included studies. When the I^2 is less than 50%, the fixed-effect model is selected. When I^2 was greater than or equal to 50%, the random effects model is selected. If the heterogeneity among studies is significant, potential sources of heterogeneity are explored through sensitivity analysis and meta-regression. Further subgroup analyses were conducted on studies that adopted different dataset types, model types, and types of predictors. Publication bias was evaluated using funnel plots and Deeks’ test.

## 3 Results

### 3.1 Study selection and study characterization

**Figure 1:**
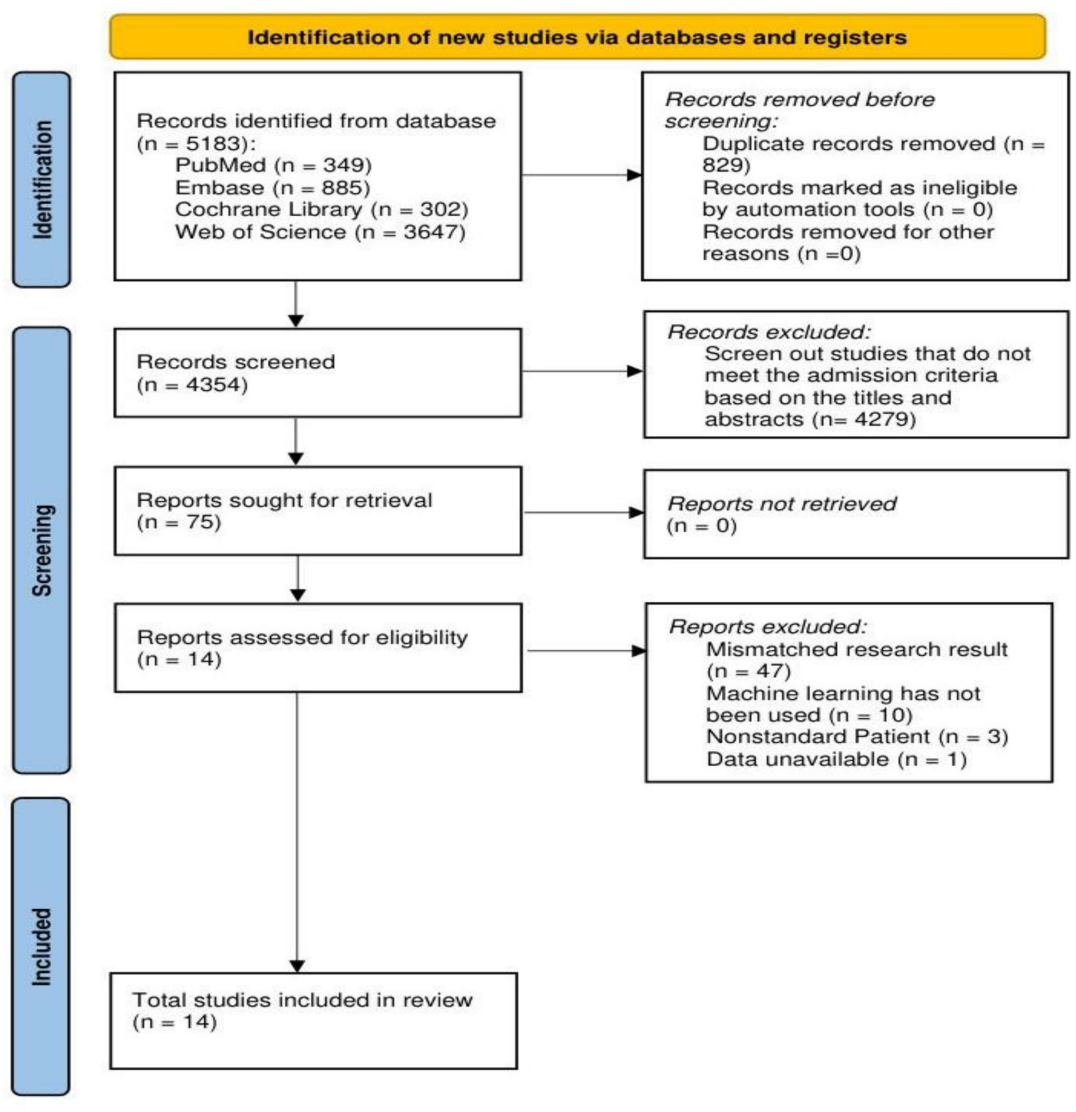
PRISMA flow chart.

According to our search strategy, a total of 5,183 relevant literatures were retrieved. After eliminating 829 duplicate literatures, a preliminary screening was conducted on the remaining 4,354 literatures. By reviewing the titles and abstracts, 4,279 literatures that did not meet the inclusion criteria were excluded. Subsequently, a full-text search and evaluation were further conducted on the remaining 75 literatures. Among them, 47 articles were excluded because the research results were not applicable to the topic of this study, 10 articles were excluded because ML methods were not used for modeling, 3 articles were excluded because the research subjects did not meet the requirements, and another 1 article was excluded because the data was unavailable. Ultimately, 14 literatures were included in the meta-analysis (10–23). The process of literature retrieval is shown in Figure 1.

Among the 14 included studies, 6 were prospective cohort studies, and the remaining 8 were retrospective cohort studies or cross-sectional studies. These studies involve four countries: Germany, Japan, South Korea and China. Among them, only one study was published in 1998, while the rest were all published after 2019. The total sample size included 16,514 patients with HZ and 2,726 patients with PHN. Regarding the definition of PHN, two studies defined it as pain that persisted for 1 month after the rash healed, 11 studies defined it as pain that persisted for 3 months after the rash healed, while one study did not clarify the definition of PHN it used. The basic information of this research can be found in Supplementary Table 3.

**Figure 2:**
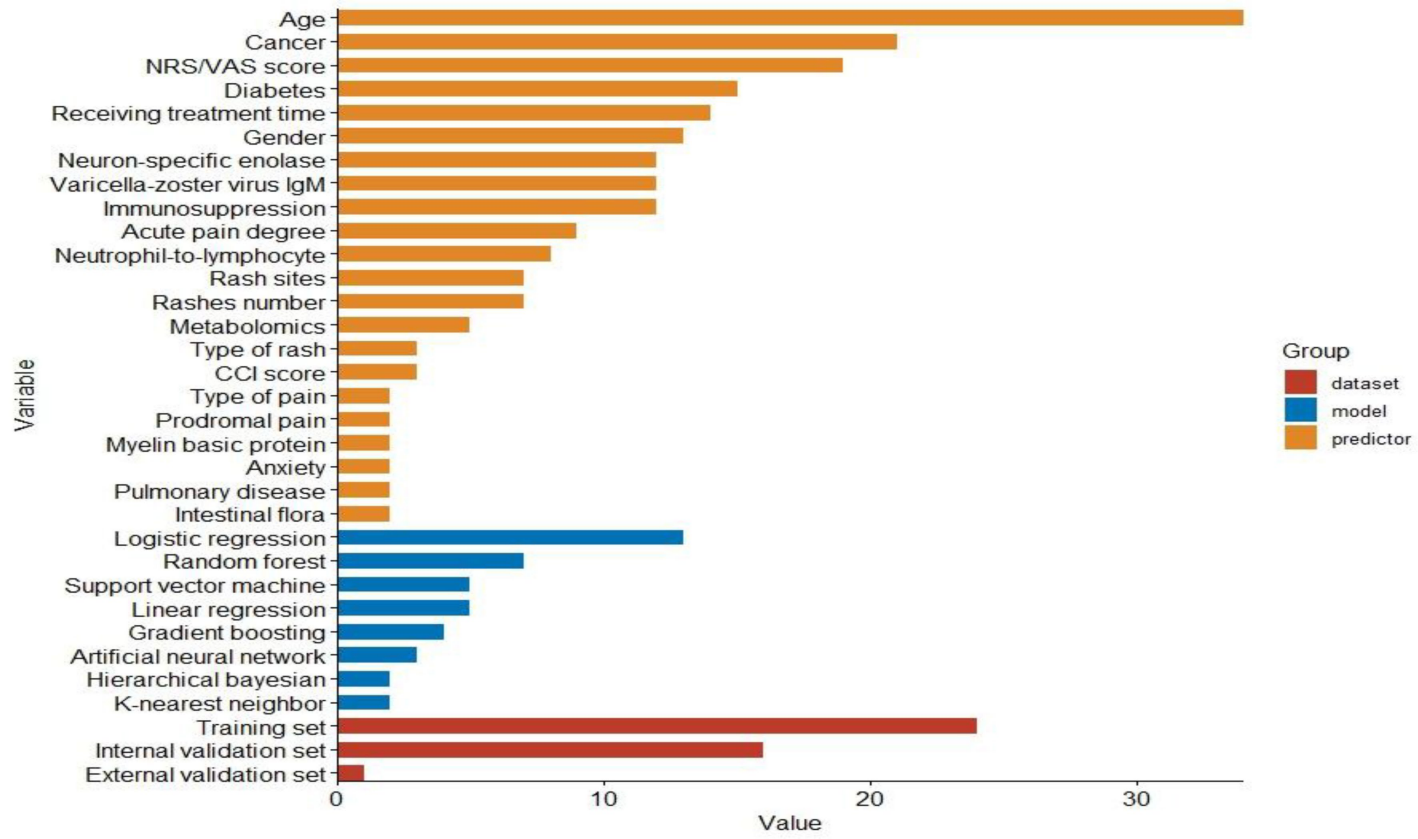
The model category, the dataset utilized, and the predictors included.

A total of 41 model performance metrics were generated in 14 studies. Among them, 24 were developed based on the training set, 16 were verified through the internal validation set, and only 1 model completed the external validation. In terms of the frequency of use of ML methods, LR (11 times) is the most common, followed by random forest (7 times), linear regression (5 times), support vector machine (5 times), gradient boosting (4 times), artificial neural network (3 times), K-nearest neighbor (2 times), and Bayesian layering (2 times). The top three in terms of the frequency of use of predictors were age (34 times), tumor (21 times), and NRS/VAS score (19 times). The summary of the characteristics of these models is presented intuitively in Figure 2.

### 3.2 Risk of bias and suitability assessment

After PROBAST’s assessment (Supplementary Table 4), all 14 studies had a certain degree of risk of bias, but most of the studies showed good applicability. Furthermore, all the studies have certain deficiencies in statistical analysis. Among them, 12 studies did not report the calculation process of the required sample size, 4 studies did not report the handling method of missing data, 7 studies did not evaluate the model according to the PROBAST standard, and 11 studies did not consider the risk of model overfitting. Besides, three studies also show obvious bias tendencies in the selection of predictors.

### 3.3 Meta-analysis of model performance

**Figure 3:**
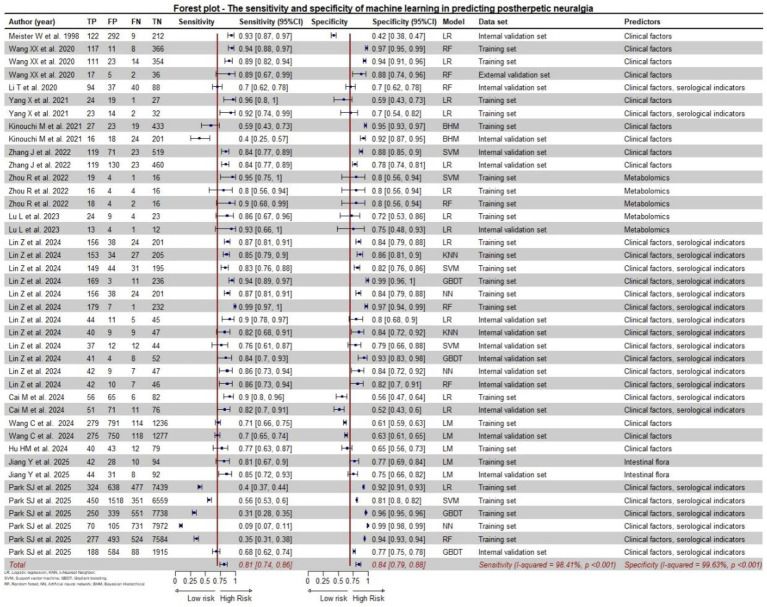
Forest plot—the sensitivity and specificity of machine learning in predicting postherpetic neuralgia.

**Figure 4:**
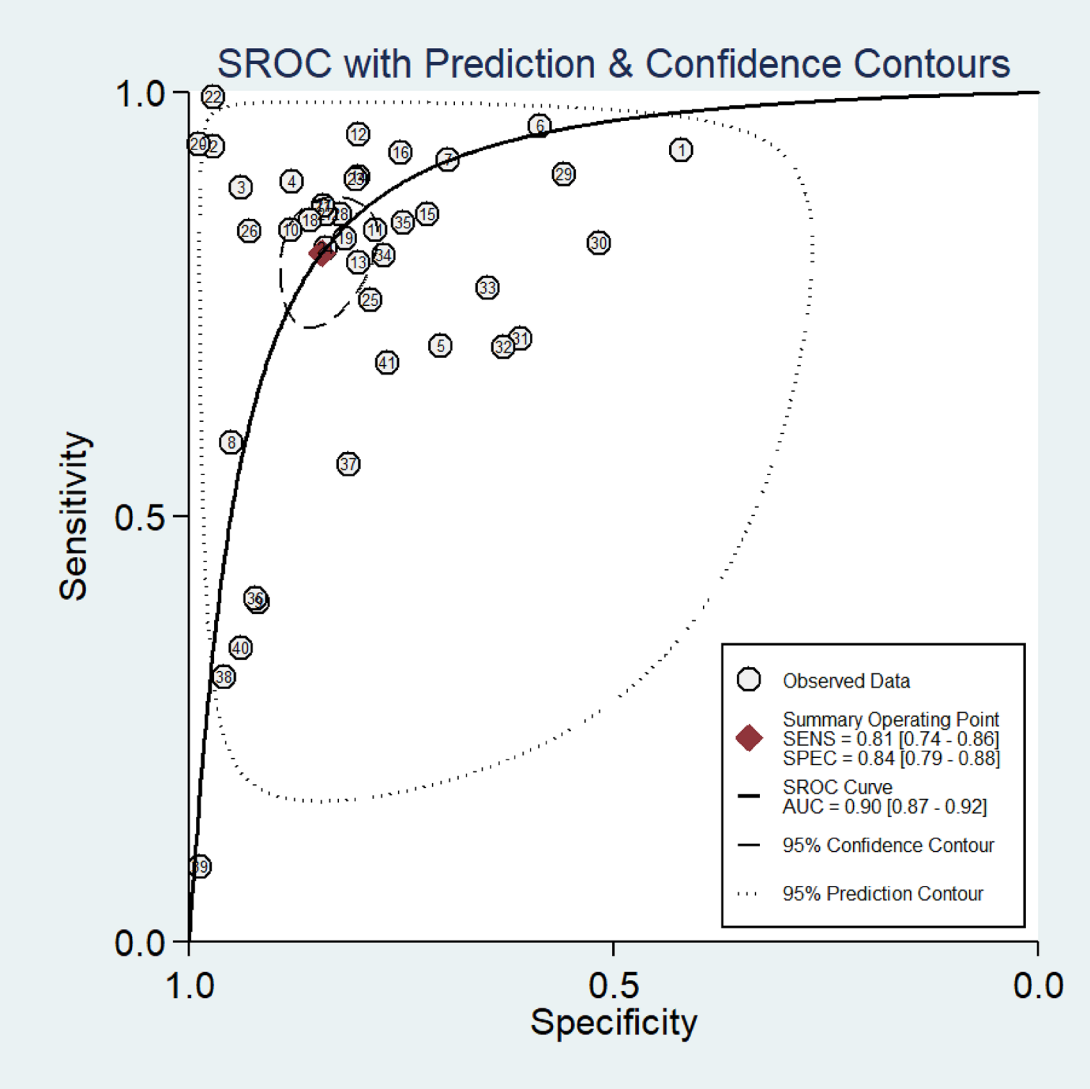
Summary ROC of the meta-analysis of predicting postherpetic neuralgia using machine learning.

We synthesized the performance of 41 models using the random effects model. The results (Figure 3) showed that the sensitivity of ML in predicting PHN was 0.81 (95% confidence interval (CI): 0.74–0.86), and the I^2 was 98.74% (*p* < 0.001). The specificity was 0.84 (95% CI: 0.79–0.88), and the I^2 was 99.63% (*p* < 0.001). Furthermore, the SROC curve (Figure 4) showed that the AUC value of ML for predicting PHN was 0.90 (95% CI: 0.87–0.92).

**Figure 5:**
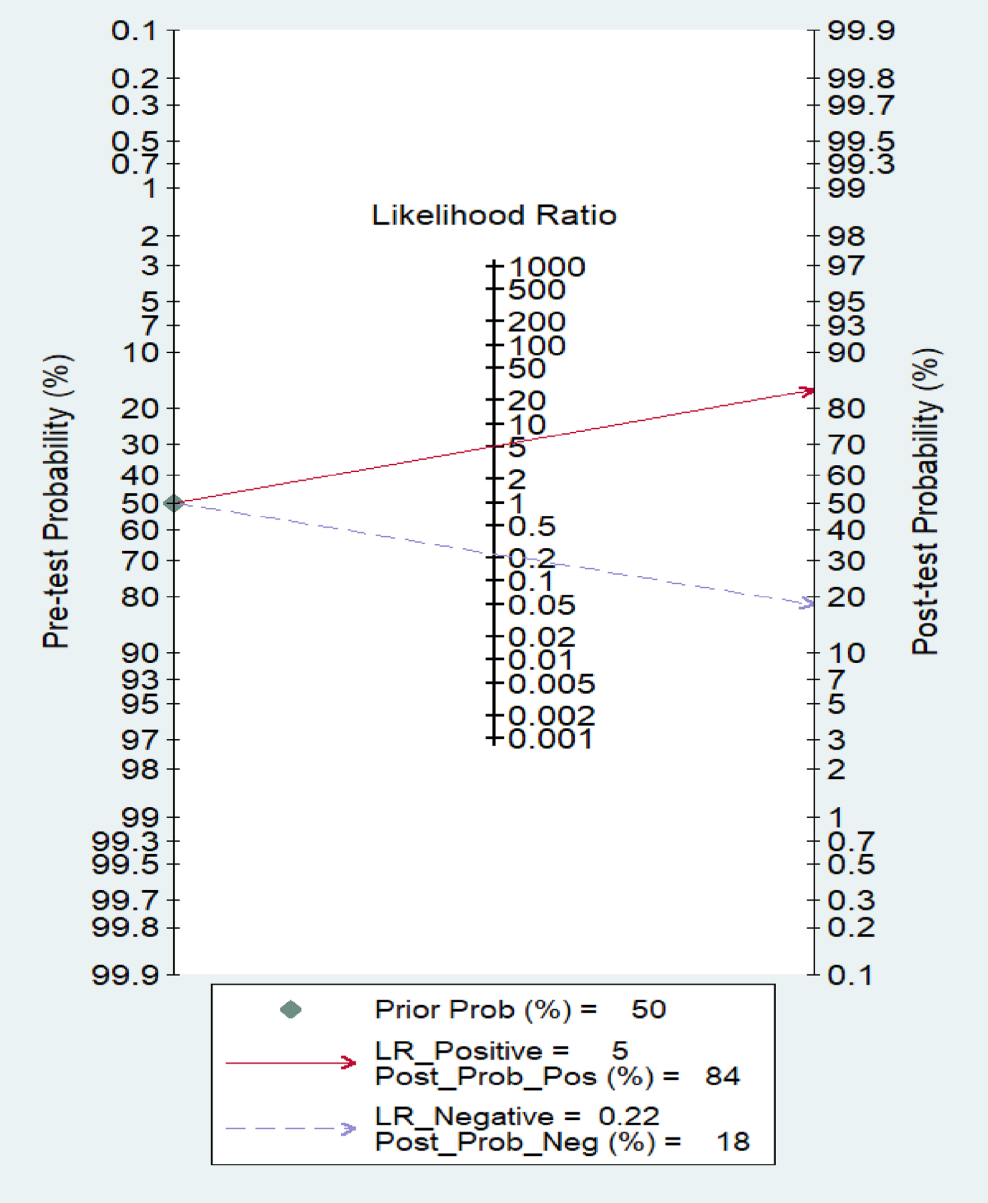
Fagan nomogram the meta-analysis of predicting postherpetic neuralgia using machine learning.

The Fagan plot (Figure 5) shows that HZ patients determined as positive by the ML model have an 80% probability of developing PHN, while the probability of HZ patients determined as negative by the model developing PHN is only 20%.

**Figure 6:**
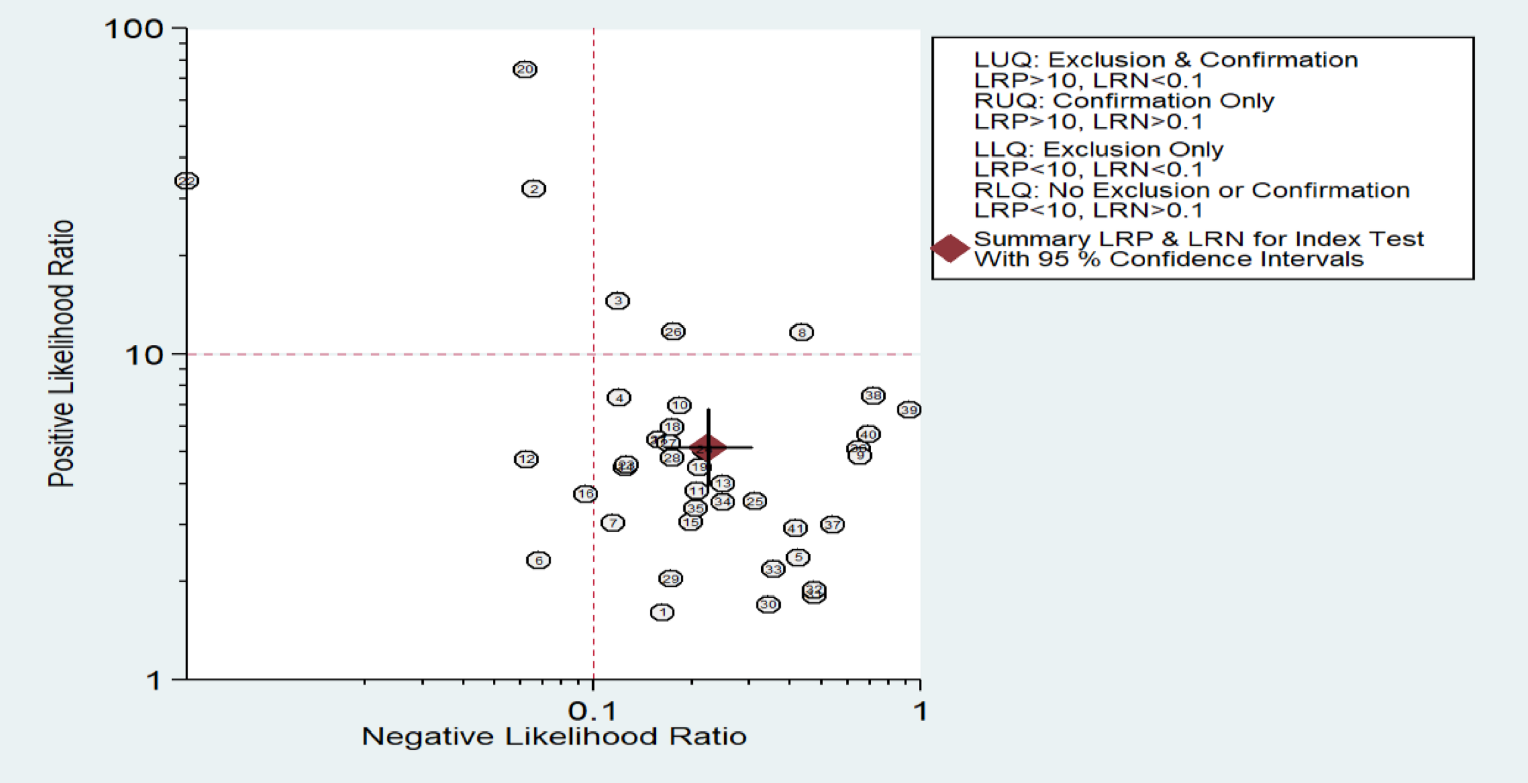
Distribution scatter diagram of the meta-analysis of predicting postherpetic neuralgia using machine learning.

The distribution scatter diagram (Figure 6) shows that the PLR of ML for predicting PHN is 5.17 [95%CI: 3.94–6.76], with a NLR of 0.22 [95%CI: 0.17–0.30].

### 3.4 Subgroup analysis

We conducted a detailed subgroup analysis, the subgroups included the comparison between the training set and the validation set, the comparison between LR and other ML methods, the comparison between clinical indicators and clinical indicators with serum indicators and omics analysis, the comparison between prospective cohort and retrospective cohort. Additionally, a subgroup was defined where PHN was defined as postherpetic pain lasting more than 3 months after HZ (Table 1).

**Table 1:** Subgroup analysis based on dataset, model, and predictor.

Subgroup analysis	Sensitivity	Specificity	Area under the curve
Dataset			
Training set	0.81 [0.70–0.89]	0.88 [0.81–0.92]	0.92 [0.89–0.94]
Validation set	0.81 [0.75–0.86]	0.78 [0.71–0.84]	0.87 [0.83–0.89]
Model			
Logistic regression	0.84 [0.78–0.88]	0.73 [0.65–0.80]	0.86 [0.83–0.89]
Other machine learning	0.78 [0.66–0.87]	0.90 [0.86–0.93]	0.93 [0.90–0.95]
Predictor			
Clinical factors	0.82 [0.72–0.89]	0.82 [0.70–0.90]	0.89 [0.86–0.91]
Add serological indicators or omics analysis	0.81 [0.72–0.89]	0.85 [0.80–0.89]	0.90 [0.87–0.92]
Cohort			
Prospective cohort	0.87 [0.82–0.90]	0.69 [0.61–0.76]	0.88 [0.84–0.90]
Retrospective cohort	0.78 [0.68–0.85]	0.88 [0.83–0.91]	0.90 [0.88–0.93]

The results of subgroup analysis showed that on the training set, the predictive performance of the ML model for PHN was sensitive (0.81 [95% CI: 0.70–0.89]), specific (0.88 [95% CI: 0.81–0.92]), and AUC (0.92 [95% CI: 0.89–0.94]; Supplementary Figure 1). On the validation set, the predictive performance of the ML model for PHN was sensitivity (0.81 [95% CI: 0.75–0.86]), specificity (0.78 [95% CI: 0.71–0.84]), and AUC (0.87 [95% CI: 0.83–0.89]; Supplementary Figure 2).

The results of subgroup analysis showed that the predictive performance of the LR model for PHN was sensitive (0.84 [95% CI: 0.78–0.88]), specific (0.73 [95% CI: 0.65–0.80]), and AUC (0.86 [95% CI: 0.83–0.89]; Supplementary Figure 3). The predictive performance of the other ML model for PHN was sensitivity (0.78 [95% CI: 0.66–0.87]), specificity (0.90 [95% CI: 0.86–0.93]), and AUC (0.93 [95% CI: 0.90–0.95]; Supplementary Figure 4).

The results of subgroup analysis showed that the predictive performance of the model constructed using clinical indicators for PHN was sensitive (0.82 [95% CI: 0.72–0.89]), specific (0.82 [95% CI: 0.70–0.90]), and AUC (0.89 [95% CI: 0.86–0.91]; Supplementary Figure 5). The predictive performance of the model constructed by comprehensively using clinical indicators, serum indicators, and omics analysis for PHN was sensitivity (0.81 [95% CI: 0.72–0.87]), specificity (0.85 [95% CI: 0.80–0.89]), and AUC (0.90 [95% CI: 0.87–0.92]; Supplementary Figure 6).

The results of subgroup analysis showed that the predictive performance of the model constructed using prospective cohort for PHN was sensitive (0.87 [95% CI: 0.82–0.90]), specific (0.69 [95% CI: 0.61–0.76]), and AUC (0.88 [95% CI: 0.84–0.90]; Supplementary Figure 7). The predictive performance of the model constructed by comprehensively using retrospective cohort for PHN was sensitivity (0.78 [95% CI: 0.68–0.85]), specificity (0.88 [95% CI: 0.83–0.91]), and AUC (0.90 [95% CI: 0.88–0.93]; Supplementary Figure 8).

The results of subgroup analysis showed that the predictive performance for PHN of the model constructed using studies which is defined PHN as pain persisting for more than 3 months after HZ was sensitive (0.81 [95% CI: 0.73–0.87]), specific (0.85 [95% CI: 0.80–0.89]), and AUC (0.90 [95% CI: 0.87–0.93]; Supplementary Figure 9).

### 3.5 Sensitivity test and meta-regression

The sensitivity test (Supplementary Figure 10) indicated that even after re-meta-analysis after removing any one study, the results remained robust.

**Figure 7:**
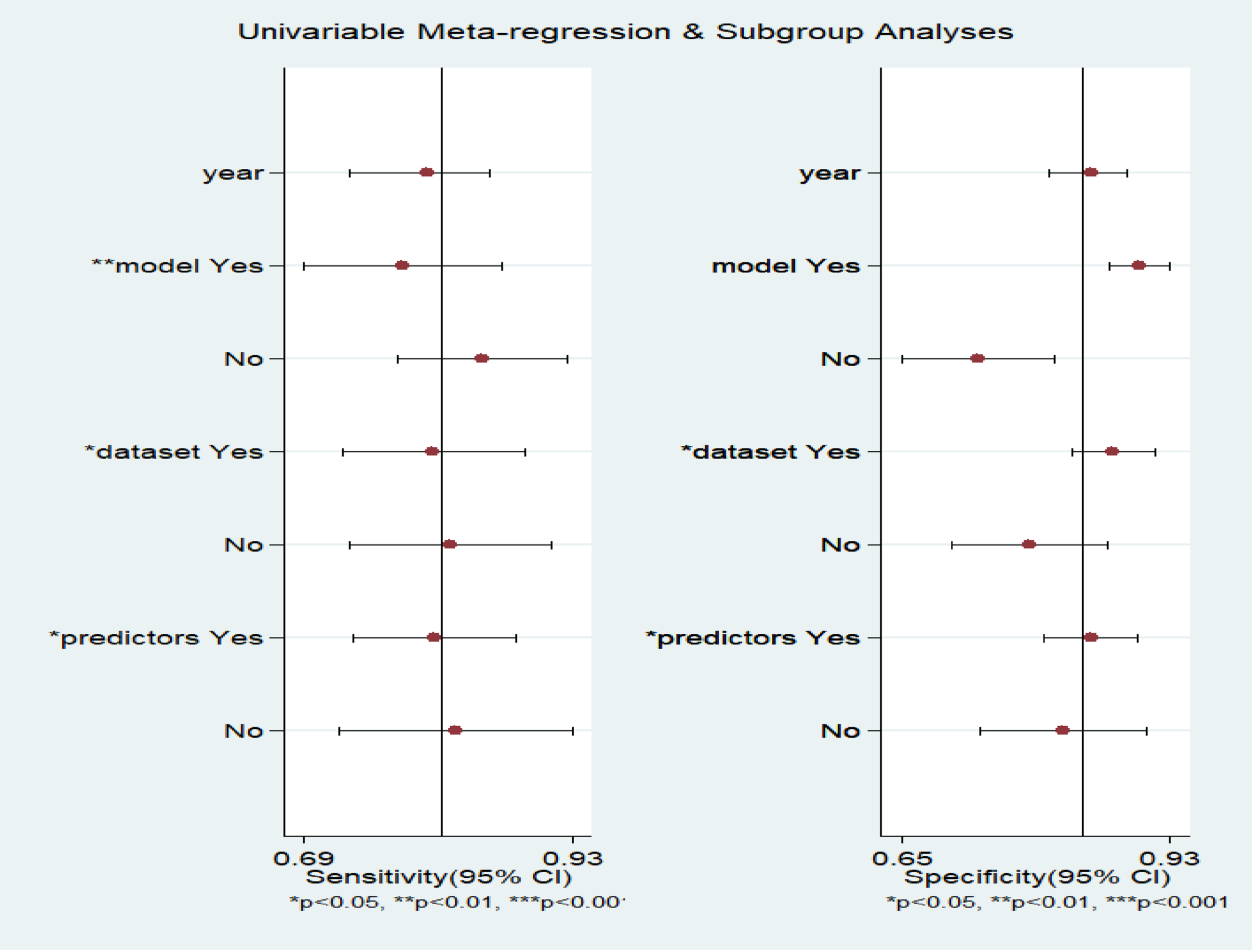
Meta regression of the meta-analysis of predicting postherpetic neuralgia using machine learning.

Meta-regression analysis (Figure 7) indicates that model type, dataset type and predictor types are the main sources leading to heterogeneity.

### 3.6 Publication bias

**Figure 8:**
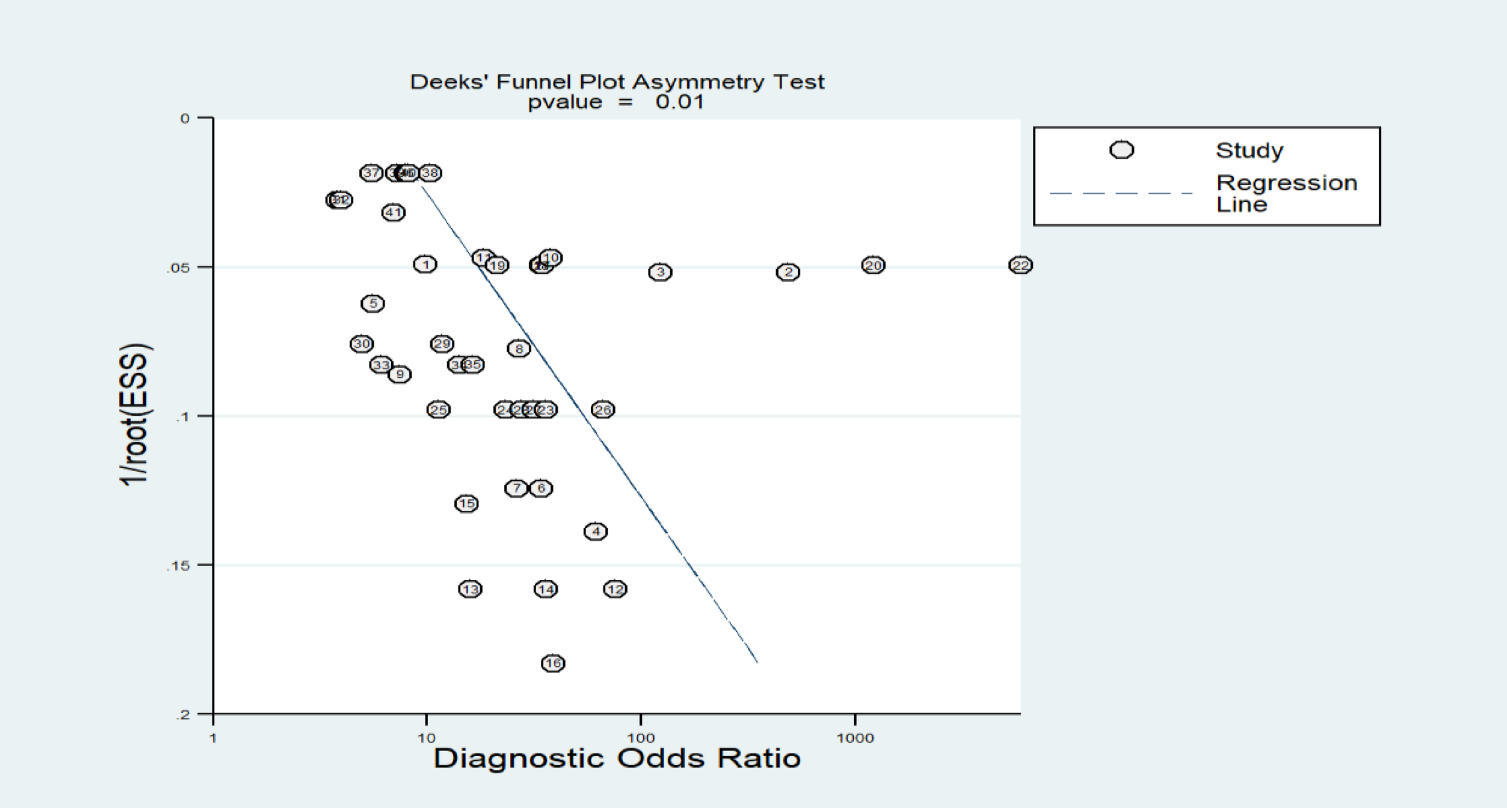
Funnel plot of the meta-analysis of predicting postherpetic neuralgia using machine learning.

The results of the Deeks’ test showed that there was a certain publication bias in this study (*p* < 0.01). Funnel plot analysis further indicates that there is asymmetry in the literature distribution on both sides of the regression line (Figure 8).

## 4 Discussion

### 4.1 Summary of meta-analysis evidence

This meta-analysis integrates the existing evidence and covers 41 models generated from 14 studies. The results show that the use of ML to predict PHN has high efficiency. The comprehensive sensitivity and specificity of ML are both greater than 0.8, which is more effective than single indicators such as age and pain score or the neuropathic pain scale (24, 25). This indicates that the precision medicine strategy based on ML has potential value in managing PHN.

### 4.2 Discussion on the selection of predictors

Among the studies included in the meta-analysis, age, pain score and tumor history were the most commonly used indicators. These three indicators have been repeatedly verified as key factors closely related to PHN (26, 27). Therefore, when developing PHN prediction models in the future, we suggest that at least these three indicators should be included in the initial screening indicator library.

Furthermore, the currently developed PHN models generally regard the pain score of patients at their first visit as an important predictor. However, the pain trajectory of HZ patients may also be of great significance for the prediction of PHN. The results of a prospective study based on the community population verified the potential value of pain trajectories in the prediction of PHN (28). The latent category trajectory model, as a method of ML, can divide heterogeneous populations into several homogeneous patterns or categories, thereby effectively describing the pain trajectories in the HZ patient group and further being used for the risk prediction of PHN (29). Therefore, we suggest that more attention be paid to the pain trajectory of patients with HZ in future research and clinical practice.

In the subgroup analysis, no significant performance differences were observed between the models constructed based on clinical indicators and those constructed in combination with clinical indicators, serum indicators or omics analysis (AUC 0.89 [95% CI: 0.86–0.91] vs. 0.90 [95% CI: 0.87–0.92]). Some indicators, although they may improve the performance of the model to a certain extent, are rarely adopted in clinical practice. This prompts us to further explore how to achieve an effective balance between model performance optimization and the complexity of predictors. The achievement of this goal requires more high-quality research for in-depth exploration.

### 4.3 Discussion on the selection of ML methods

Among the included literature, LR is widely used as the main ML method. This might be attributed to its relatively simple structure, which is easy to understand and more acceptable to medical professionals (30). A meta-analysis summarized the differences in predictive efficacy between LR and other ML methods, and pointed out that LR has similar predictive performance to other ML methods (31). In this study, we found that in the field of predicting PHN, the predictive efficacy of the LR model was generally comparable to that of other ML methods. Among them, the LR model shows higher sensitivity (0.84 [95% CI: 0.78–0.88] vs. 0.78 [95% CI: 0.66–0.87]), while other ML models demonstrate better specificity (0.90 [95% CI: 0.86–0.93] vs. 0.73 [95% CI: 0.65–0.80]). Therefore, on the premise of fully explaining the model, the performance advantages of other ML models over LR models in the field of predicting PHN remain a highly controversial topic.

Ensemble techniques typically integrate multiple ML algorithms through methods such as bagging, boosting, stacking, and voting, thereby balancing the strengths and weaknesses of ML (32). We suggest that if the constructed PHN ML models have their own advantages and disadvantages in terms of performance indicators, the ensemble learning method can be attempted to be adopted to integrate the advantages of different ML algorithms, thereby further improving the overall performance of the models.

### 4.4 Discussion on model overfitting and the balance between sensitivity and specificity

In the subgroup analysis of the meta-analysis, we observed that the performance of the ML model on the training set was significantly higher than that on the test set (AUC 0.92 [95% CI: 0.89–0.94] vs. 0.87 [95% CI: 0.83–0.89]). This result indicates that the currently published PHN prediction models may have a certain degree of overfitting risk. Meanwhile, in the validation set, the specificity of predicting PHN decreased significantly (0.88 [95% CI: 0.81–0.92] vs. 0.78 [95% CI: 0.71–0.84]). This means that when applied to external datasets, the PHN prediction model has a certain false positive rate. Furthermore, most studies do not adopt external validation methods, which leads to considerable uncertainty in the performance evaluation of these models on external data. Therefore, in the future development process of PHN models, we suggest comprehensively applying strategies such as external validation, cross-validation, and resampling. These methods have been widely proven to significantly enhance the generalization ability of the model (33).

Besides, we observed that the models constructed based on prospective cohorts performed worse than those based on retrospective cohorts (AUC 0.88 [95% CI: 0.84–0.90] vs. 0.90 [95% CI: 0.88–0.93]). This might be because the risk of bias in retrospective data is higher, and thus the risk of overfitting in the models built from retrospective data is also higher. Therefore, we suggest that future research should, as far as possible, adopt the strategy of prospective cohort studies.

We have noticed that in the process of model development, many researchers often face the problem of balancing sensitivity and specificity (34). For instance, in the development of the PHN ML model, a study from South Korea reported that their model demonstrated low sensitivity (<0.6) and high specificity (>0.9) (23). In fact, based on the ROC curve drawn in this study, researchers were fully able to achieve a balance between sensitivity and specificity by adjusting the cut-off value. To solve this problem, we suggest that in the future when developing PHN prediction models, at least three cutoff values should be reported to meet different clinical needs. The first one is the balanced cutoff value, which is used to determine whether PHN occurs. The second one is called the positive cutoff value. This cutoff value should ensure that the sensitivity is higher than 90% without overly focusing on specificity. The third one is called the negative cutoff value. This cutoff value should ensure that the specificity is higher than 90% without overly focusing on the sensitivity. By introducing these three cut-off values, more comprehensive support can be provided for clinical decision-making.

### 4.5 Discussion on the clinical application of machine learning in PHN prediction

In recent years, numerous advanced pain management techniques, such as spinal cord stimulation and stellate ganglion block, have demonstrated efficacy in preventing the onset of PHN (35–37). When integrated with efficient machine learning models, these interventions can generate synergistic effects. HZ patients identified by machine learning models as being at high risk for developing PHN can be prioritized for advanced pain management strategies to reduce the likelihood of PHN occurrence, whereas those classified as low-risk can be managed with conventional treatment approaches. This integrated approach enables optimal patient care in a cost-effective manner.

However, despite the good performance demonstrated by current PHN prediction models, their applicability to external datasets remains uncertain due to the absence of external validation, non-standardized reporting formats, and limited sample sizes used in model development. We suggest that future development of PHN prediction models should involve at least two independent cohorts and strictly follow the TRIPOD guidelines to ensure standardized reporting of predictive models (38).

### 4.6 Limitations

Despite rigorous search and systematic evaluation, this meta-analysis still has several limitations that cannot be completely avoided. Firstly, the heterogeneity among the included studies was relatively high. The difference between ML models and traditional diagnostic tests lies in the different built-in parameters and cutoff values of each model. ML is more sensitive and more susceptible to the influence of data quality, which leads to significant heterogeneity among ML models. The meta-regression indicated that this might stem from differences in datasets, model selection, and predictor definitions. In the future, this issue can be addressed by adopting a more rigorous standardized reporting framework for predictive models and using larger sample sizes to improve model quality. Secondly, some studies did not strictly follow the standard norms of the prediction model reports, and the vast majority of studies lacked external validation (39). Therefore, a cautious attitude should be maintained when interpreting the performance of these studies. Finally, the included studies showed significant publication bias, which might lead to a certain degree of overestimation of the conclusions regarding ML performance in this study. More high-quality research is urgently needed in the future to further verify and supplement the current findings.

## 5 Conclusion

ML is a promising tool for predicting PHN. The PHN prediction model based on ML shows high prediction accuracy and performs better than a single indicator or traditional scales. However, most models generally face problems such as the lack of external validation, the existence of overfitting phenomena, and insufficient reporting standardization. This has raised concerns regarding the ability of the PHN prediction model to maintain high accuracy when applied to external populations. In the future development of PHN models, we recommend implementing strict external validation, clearly reporting balanced cutoff values, positive cutoff values, and negative cutoff values, and adhering to international norms for predictive model reporting (such as the TRIPOD guidelines). Meanwhile, when necessary, introduce ensemble learning methods and pain trajectory analysis. The aim is to further improve the generalization ability and practical application value of the model.

## Data Availability

Publicly available datasets were analyzed in this study. This data can be found at: the data used in this article can all be retrieved in PubMed, Web of Science, Embase and Cochrane Library.
