# Supplementary material for: Evaluating the efficacy of machine learning in predicting postherpetic neuralgia: a systematic review and meta-analysis
Source: Front Neurol. 2025 Sep 15;16:1632682. doi: 10.3389/fneur.2025.1632682 (PMC12476998; doi:10.3389/fneur.2025.1632682)
Supplement: Supplementary file 1 [file Data_Sheet_1.pdf]

Supplementary tables and figures Contents

Supplementary Tables

1. Supplementary Table 1 PRISMA checklist--2

2. Supplementary Table 2 Search strategies---5

3. Supplementary Table 3 Basic information of included studies--6

4. Supplementary Table 4 The Prediction model risk of bias assessment tool (PROBAST) -------------------------------8

Supplemental Figures

5. Supplemental Figure 1 Subgroup analysis - Based on the data (training set) ---10

6. Supplemental Figure 2 Subgroup analysis - Based on the data (validation set) -------------------------------------- 10

7. Supplemental Figure 3 Subgroup analysis - Based on the model (Logistic regression model) -------------------- 10

8. Supplemental Figure 4 Subgroup analysis - Based on the model (other machine learning models) -------------11

9. Supplemental Figure 5 Subgroup analysis - Based on the predictor (clinical factors) --------------------------------11

10. Supplemental Figure 6 Subgroup analysis - Based on the predictor (clinical factors and serological indicators or omics analysis) --11

11. Supplemental Figure 7 Subgroup analysis - Based on the cohort (prospective)-------------------------------------12

12. Supplemental Figure 8 Subgroup analysis - Based on the cohort (retrospective) -----------------------------------12

13. Supplemental Figure 9 Subgroup analysis - Based on the definition of PHN (three months) --------------------12

14. Supplemental Figure 10 Sensitivity analysis ---13

Other

12. Reference--14

Supplementary Table 1 PRISMA checklist

Section and Topic	Item #	Checklist item	Location where item is reported
**TITLE**			
Title	1	Identify the report as a systematic review.	Title
**ABSTRACT**			
Abstract	2	See the PRISMA 2020 for Abstracts checklist.	Abstract
**INTRODUCTION**			
Rationale	3	Describe the rationale for the review in the context of existing knowledge.	Introduction, paragraph three
Objectives	4	Provide an explicit statement of the objective(s) or question(s) the review addresses.	Introduction, paragraph four
**METHODS**			
Eligibility criteria	5	Specify the inclusion and exclusion criteria for the review and how studies were grouped for the syntheses.	Methods, 2.3
Information sources	6	Specify all databases, registers, websites, organisations, reference lists and other sources searched or consulted to identify studies. Specify the date when each source was last searched or consulted.	Methods, 2.2
Search strategy	7	Present the full search strategies for all databases, registers and websites, including any filters and limits used.	Methods, 2.2 and Supplementary Table 2
Selection process	8	Specify the methods used to decide whether a study met the inclusion criteria of the review, including how many reviewers screened each record and each report retrieved, whether they worked independently, and if applicable, details of automation tools used in the process.	Methods, 2.4
Data collection process	9	Specify the methods used to collect data from reports, including how many reviewers collected data from each report, whether they worked independently, any processes for obtaining or confirming data from study investigators, and if applicable, details of automation tools used in the process.	Methods, 2.4
Data items	10a	List and define all outcomes for which data were sought. Specify whether all results that were compatible with each outcome domain in each study were sought (e.g. for all measures, time points, analyses), and if not, the methods used to decide which results to collect.	Methods, 2.4
	10b	List and define all other variables for which data were sought (e.g. participant and intervention characteristics, funding sources). Describe any assumptions made about any missing or unclear information.	Methods, 2.4
Study risk of bias assessment	11	Specify the methods used to assess risk of bias in the included studies, including details of the tool(s) used, how many reviewers assessed each study and whether they worked independently, and if applicable, details of automation tools used in the process.	Methods, 2.5

Section and Topic	Item #	Checklist item	Location where item is reported
Effect measures	12	Specify for each outcome the effect measure(s) (e.g. risk ratio, mean difference) used in the synthesis or presentation of results.	Methods, 2.6
Synthesis methods	13a	Describe the processes used to decide which studies were eligible for each synthesis (e.g. tabulating the study intervention characteristics and comparing against the planned groups for each synthesis (item #5)).	Methods, 2.6
	13b	Describe any methods required to prepare the data for presentation or synthesis, such as handling of missing summary statistics, or data conversions.	Methods, 2.6
	13c	Describe any methods used to tabulate or visually display results of individual studies and syntheses.	Methods, 2.6
	13d	Describe any methods used to synthesize results and provide a rationale for the choice(s). If meta-analysis was performed, describe the model(s), method(s) to identify the presence and extent of statistical heterogeneity, and software package(s) used.	Methods, 2.6
	13e	Describe any methods used to explore possible causes of heterogeneity among study results (e.g. subgroup analysis, meta-regression).	Methods, 2.6
	13f	Describe any sensitivity analyses conducted to assess robustness of the synthesized results.	Methods, 2.6
Reporting bias assessment	14	Describe any methods used to assess risk of bias due to missing results in a synthesis (arising from reporting biases).	Methods, 2.6
Certainty assessment	15	Describe any methods used to assess certainty (or confidence) in the body of evidence for an outcome.	Methods, 2.6
**RESULTS**			
Study selection	16a	Describe the results of the search and selection process, from the number of records identified in the search to the number of studies included in the review, ideally using a flow diagram.	Results, 3.1
	16b	Cite studies that might appear to meet the inclusion criteria, but which were excluded, and explain why they were excluded.	Results, 3.1
Study characteristics	17	Cite each included study and present its characteristics.	Results, 3.1
Risk of bias in studies	18	Present assessments of risk of bias for each included study.	Results, 3.2
Results of individual studies	19	For all outcomes, present, for each study: (a) summary statistics for each group (where appropriate) and (b) an effect estimate and its precision (e.g. confidence/credible interval), ideally using structured tables or plots.	Results, 3.3 and figure 3
Results of syntheses	20a	For each synthesis, briefly summarise the characteristics and risk of bias among contributing studies.	Results, 3.3
	20b	Present results of all statistical syntheses conducted. If meta-analysis was done, present for each the summary estimate and its precision (e.g. confidence/credible interval) and measures of statistical heterogeneity. If comparing groups, describe the direction of the effect.	Results, 3.3
	20c	Present results of all investigations of possible causes of heterogeneity among study results.	Results, 3.3

Section and Topic	Item #	Checklist item	Location where item is reported
	20d	Present results of all sensitivity analyses conducted to assess the robustness of the synthesized results.	Results, 3.3
Reporting biases	21	Present assessments of risk of bias due to missing results (arising from reporting biases) for each synthesis assessed.	Results, 3.6
Certainty of evidence	22	Present assessments of certainty (or confidence) in the body of evidence for each outcome assessed.	Results, 3.4 and 3.5
**DISCUSSION**			
Discussion	23a	Provide a general interpretation of the results in the context of other evidence.	Discussion, 4.1 and 4.5
	23b	Discuss any limitations of the evidence included in the review.	Discussion, 4.1 and 4.5
	23c	Discuss any limitations of the review processes used.	Discussion, 4.1 and 4.5
	23d	Discuss implications of the results for practice, policy, and future research.	Conclusion
**OTHER INFORMATION**			
Registration and protocol	24a	Provide registration information for the review, including register name and registration number, or state that the review was not registered.	Ethics statement
	24b	Indicate where the review protocol can be accessed, or state that a protocol was not prepared.	Ethics statement
	24c	Describe and explain any amendments to information provided at registration or in the protocol.	Ethics statement
Support	25	Describe sources of financial or non-financial support for the review, and the role of the funders or sponsors in the review.	Funding
Competing interests	26	Declare any competing interests of review authors.	Conflict of Interest
Availability of data, code and other materials	27	Report which of the following are publicly available and where they can be found: template data collection forms; data extracted from included studies; data used for all analyses; analytic code; any other materials used in the review.	Data Availability Statement

Supplementary Table 2 Search strategies

Pubmed 2025/05/06: 349 results

NO.	Retrieval strategy	Results
**1**	"Neuralgia, Postherpetic" [Mesh] OR "PHN" [Title/Abstract] OR "postherpetic neuralgia"[Title/Abstract] OR "post herpetic neuralgia"[Title/Abstract] OR "post-herpetic neuralgia"[Title/Abstract] OR "postherpetic pain"[Title/Abstract] OR "post herpetic pain"[Title/Abstract] OR post-herpetic pain[Title/Abstract] OR (("Neuralgia"[Mesh] OR "Pain"[Mesh] OR Neuralgia[Title/Abstract] OR Pain[Title/Abstract]) AND ("Herpes Zoster"[Mesh] OR zoster[Title/Abstract] OR shingles[Title/Abstract] OR zona[Title/Abstract] OR VZV[Title/Abstract]))	7,304
**2**	"Logistic Model"[Title/Abstract] OR "predict"[Title/Abstract] OR "Prediction" [Title/Abstract] OR "Machine learning" [Title/Abstract] OR "Model" [Title/Abstract] OR "ROC" [Title/Abstract] OR "Area under the curve" [Title/Abstract] OR "AUC" [Title/Abstract]	2,491,318
**3**	1 AND 2	405
**4**	"Animals"[Mesh] NOT "Humans"	5,241,633
**5**	3 NOT 4	349

Web of science 2025/05/06: 3,647 results

NO.	Retrieval strategy	Results
**1**	TS=（"Neuralgia, Postherpetic" OR "PHN" OR "postherpetic neuralgia" OR "post herpetic neuralgia" OR "post-herpetic neuralgia" OR "postherpetic pain" OR "post herpetic pain" OR "post-herpetic pain" OR "zoster" OR "shingles" OR "VZV"）	62,375
**2**	TS= ("Logistic Model" OR "predict" OR " Prediction" OR "Machine learning" OR "Model" OR "ROC" OR "Area under the curve" OR "AUC")	12,209,728
**3**	1 AND 2	3,647

Embase 2025/05/06: 885 results

NO.	Retrieval strategy	Results
**1**	'postherpetic neuralgia'/exp OR 'phn':ti,ab,kw OR 'postherpetic neuralgia':ti,ab,kw OR 'post herpetic neuralgia':ti,ab,kw OR 'post-herpetic neuralgia':ti,ab,kw OR 'postherpetic pain':ti,ab,kw OR 'post herpetic pain':ti,ab,kw OR 'post-herpetic pain':ti,ab,kw OR (('neuralgia'/exp OR 'pain'/exp OR 'neuralgia':ti,ab,kw OR 'pain':ti,ab,kw) AND ('herpes zoster'/exp OR 'zoster':ti,ab,kw OR 'shingles':ti,ab,kw OR 'zona':ti,ab,kw OR 'vzv':ti,ab,kw))	20,033
**2**	'Logistic Model':ti,ab,kw OR 'predict':ti,ab,kw OR 'Prediction':ti,ab,kw OR 'Machine learning':ti,ab,kw OR 'Model':ti,ab,kw OR 'ROC':ti,ab,kw OR 'Area under the curve':ti,ab,kw OR 'AUC':ti,ab,kw	3,278,520
**3**	1 AND 2	885

Cochrane 2025/05/06: 302 results

NO.	Retrieval strategy	Results
**1**	MeSH descriptor: [Neuralgia, Postherpetic] explode all trees	1,385
**2**	PHN OR postherpetic neuralgia OR post herpetic neuralgia OR (post-herpetic neuralgia) OR postherpetic pain OR post herpetic pain OR (post-herpetic pain)	1,663
**3**	1 OR 2	1,663
**4**	MeSH descriptor: [Neuralgia] explode all trees	4,146

**5**	MeSH descriptor: [Pain] explode all trees	275,625
**6**	4 OR 5	276,218
**7**	MeSH descriptor: [Herpes Zoster] explode all trees	2,917
**8**	zoster OR shingles OR zona OR VZV	3,768
**9**	7 OR 8	3,768
**10**	6 AND 9	1,347
**11**	3 OR 10	2,326
**12**	MeSH descriptor: [Logistic Model] explode all trees	9,729
**13**	MeSH descriptor: [predict] explode all trees	29,247
**14**	MeSH descriptor: [Prediction] explode all trees	18,883
**15**	MeSH descriptor: [Machine learning] explode all trees	3,682
**16**	MeSH descriptor: [Model] explode all trees	132,074
**17**	MeSH descriptor: [ROC] explode all trees	6,742
**18**	MeSH descriptor: [Area under the curve] explode all trees	36,981
**19**	MeSH descriptor: [AUC] explode all trees	25,153
**20**	12 OR 13 OR 14 OR 15 OR 16 OR 17 OR 18 OR 19	201,707
**21**	11 AND 20	302

Supplementary Table 3 Basic information of included studies

Author	Year	Country	Research type	Sample size (HZ)	Sample size (PHN)	Criterion for HZ	Criterion for PHN	Model type	Machine learning method	Predictors
**Meister W et al.(1)**	1998	Germany	Prospective	635	131	Diagnosis based on clinical symptoms	Persistent pain for one month after the rash scabbed over	A	LR	Female, age, number of lesions, rash in the lumbosacral region, hemorrhagic lesion, prodromal pain
**Wang XX et al.(2)**	2020	China	Retrospective	562	144	Unclear	Persistent pain for three months after the rash scabbed over	B	LR, RF	Age, NRS score, rash site, CCI score, antiviral therapy, immunosuppression
**Li T et al.(3)**	2020	China	Retrospective	1303	571	Diagnosis based on clinical symptoms	Persistent pain for one month after the rash scabbed over	A	RF	2958 indicators
**Yang X et al.(4)**	2021	China	Prospective	71	25	Diagnosis based on clinical symptoms	Persistent pain for three months after the rash scabbed over	A	LR	Age, acute pain, response to treatment drugs, level of myelin basic protein
**Kinouchi M et al.(5)**	2021	Japan	Retrospective	761	86	Diagnosis based on clinical symptoms	Persistent pain for three months after the rash scabbed over	B	BHM	Severity of the rash, age
**Zhang J et al.(6)**	2022	China	Retrospective	732	142	Diagnosis based on clinical symptoms	Unclear	A	LR, SVM	Gender, age, VAS scores, skin lesion area, initial treatment time, anxiety, sites of rash, type of rash, types of pain
**Zhou R et al.(7)**	2022	China	Prospective	40	20	Diagnosis based on clinical symptoms	Persistent pain for three months after the rash scabbed over	A	LR, RF, SVM	Metabolomics
**Lu L et al.(8)**	2023	China	Prospective	90	42	Diagnosis based on clinical symptoms	Persistent pain for three months after the rash scabbed over	A	LR	Metabolomics
**Lin Z et al.(9)**	2024	China	Retrospective	524	229	Diagnosis based on clinical symptoms	Persistent pain for three months after the rash scabbed over	A	LR, KNN, SVM, RF, GBDT, NN	Age, NRS, rash recovery time, receiving treatment time, history of malignancy, diabetes, varicella-zoster virus lgM, Neuron-specific enolase-cific enolase
**Cai M et al.(10)**	2024	China	Retrospective	209	62	Diagnosis based on clinical symptoms	Persistent pain for three months after the rash scabbed over	A	LR	Age, NRS, platelet-to-lymphocyte ratio
**Wang C et al. (11)**	2024	China	Retrospective	2420	393	Diagnosis based on clinical symptoms	Persistent pain for three months after the rash scabbed over	A	LM	Gender, age, diabetes mellitus, pulmonary disease, malignant tumors, connective tissue diseases
**Hu HM et al.(12)**	2024	China	Prospective	174	52	Diagnosis based on clinical symptoms	Persistent pain for three months after the rash scabbed over	A	LM	Gender, age, prodromal pain, rash area, pain severity

**Jiang Y et al.(13)**	2025	China	Prospective	115	28	Diagnosis based on clinical symptoms	Persistent pain for three months after the rash scabbed over	A	LM	Intestinal flora
**Park SJ et al.(14)**	2025	Korea	Retrospective	8878	801	Diagnosis based on clinical symptoms	Persistent pain for three months after the rash scabbed over	A	LR, SVM, RF, GBDT, NN	Gender, age, history of shingles and cancer, Immunosuppression, pain severity, neutrophil--to--lymphocyte

Abbreviation: A, only develop models or only conduct internal verification; B, the model was developed and externally verified; HZ, herpes zoster; PHN, postherpetic neuralgia; LR, logistic regression; LM, linear regression; KNN, k-nearest neighbor; SVM, support vector machine; GBDT, gradient boosting; RF, random forest; NN, artificial neural network; BHM, hierarchical bayesian.

Supplementary Table 4 The Prediction model risk of bias assessment tool (PROBAST) of the included studies

Author	Year	Risk of bias				Applicability			Overall	
		Participants	Predictors	Outcome	Analysis	Participants	Predictors	Outcome	Risk of bias	Applicability
**Meister W et al.(1)**	1998	+	+	+	-	+	+	+	-	+
**Wang XX et al.(2)**	2020	+	-	+	-	+	+	+	-	+
**Li T et al.(3)**	2020	+	-	+	-	+	+	+	-	+
**Yang X et al.(4)**	2021	-	-	+	-	+	+	+	-	+
**Kinouchi M et al.(5)**	2021	+	+	+	-	+	+	+	-	+
**Zhang J et al.(6)**	2022	-	+	+	-	+	+	-	-	-
**Zhou R et al.(7)**	2022	-	+	+	-	+	+	+	-	+
**Lu L et al.(8)**	2023	-	+	+	-	+	+	+	-	+

**Lin Z et al.(9)**	2024	-	+	+	-	+	+	+	-	+
**Cai M et al.(10)**	2024	-	+	+	-	+	+	+	-	+
**Wang C et al. (11)**	2024	-	-	+	-	+	+	+	-	+
**Hu HM et al.(12)**	2024	+	+	+	-	+	+	+	-	+
**Jiang Y et al.(13)**	2025	+	+	+	-	+	+	+	-	+
**Park SJ et al.(14)**	2025	-	+	+	-	+	+	+	-	+

Supplemental Figure 1 Subgroup analysis - Based on the data (training set)

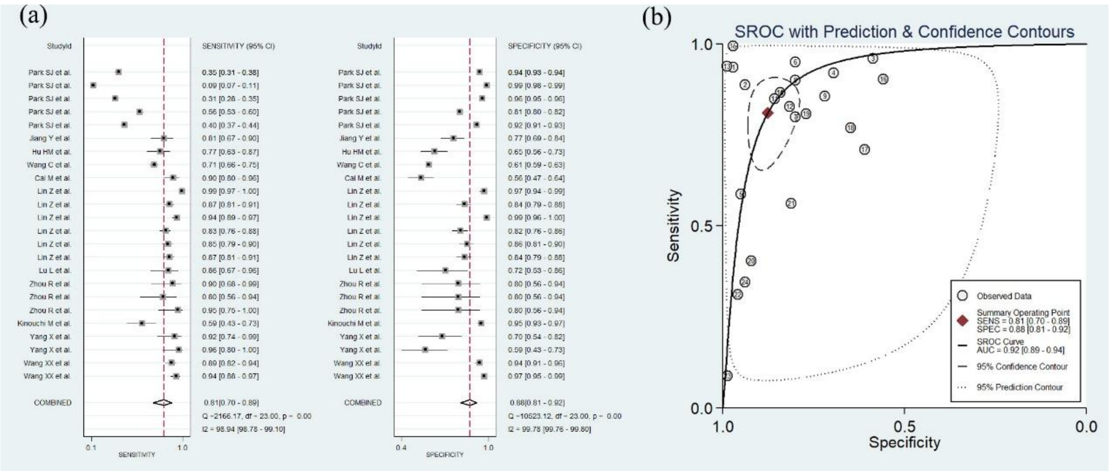

Supplemental Figure 2 Subgroup analysis - Based on the data (validation set)

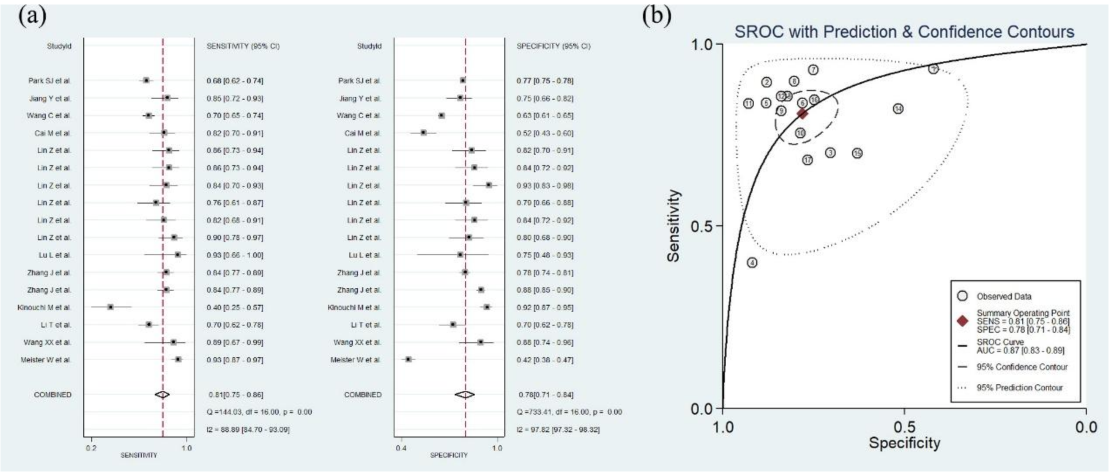

Supplemental Figure 3 Subgroup analysis - Based on the model (Logistic regression model)

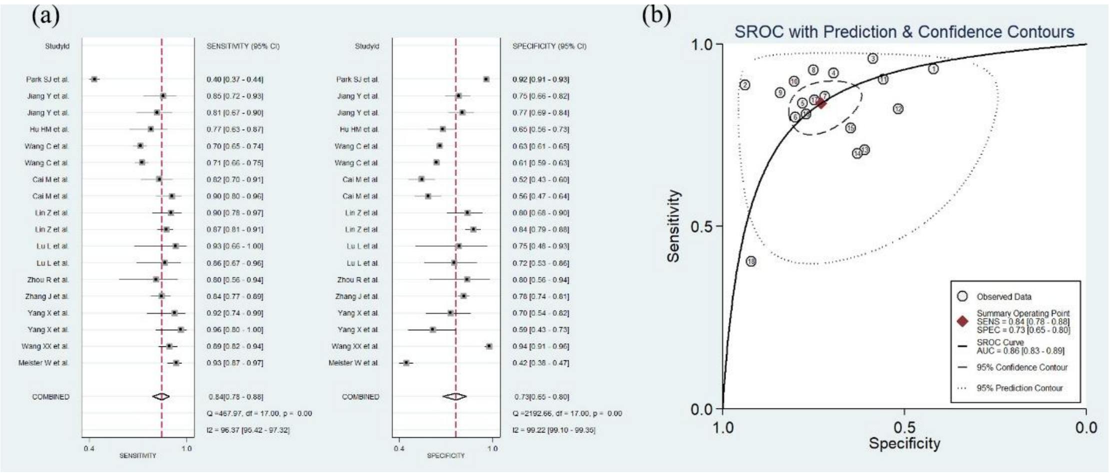

Supplemental Figure 4 Subgroup analysis - Based on the model (other machine learning models)

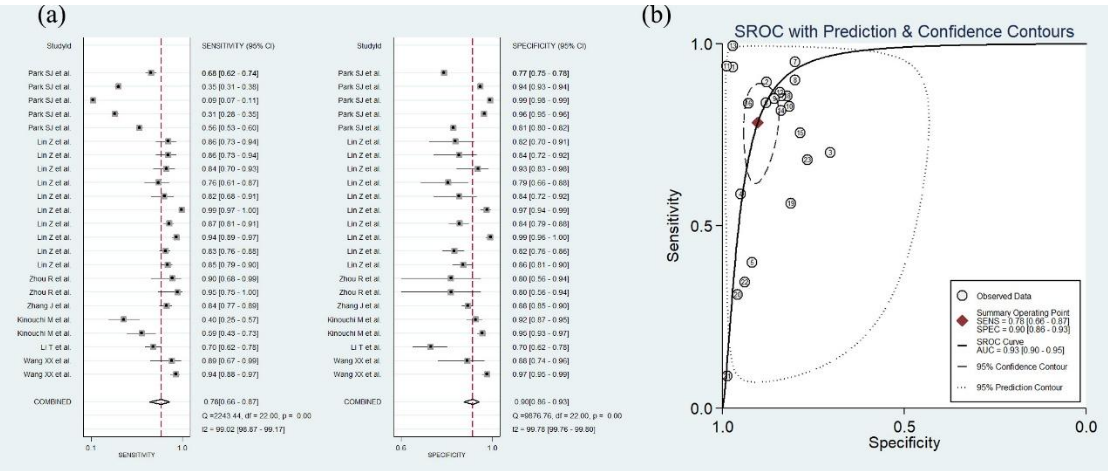

Supplemental Figure 5 Subgroup analysis - Based on the predictor (clinical factors)

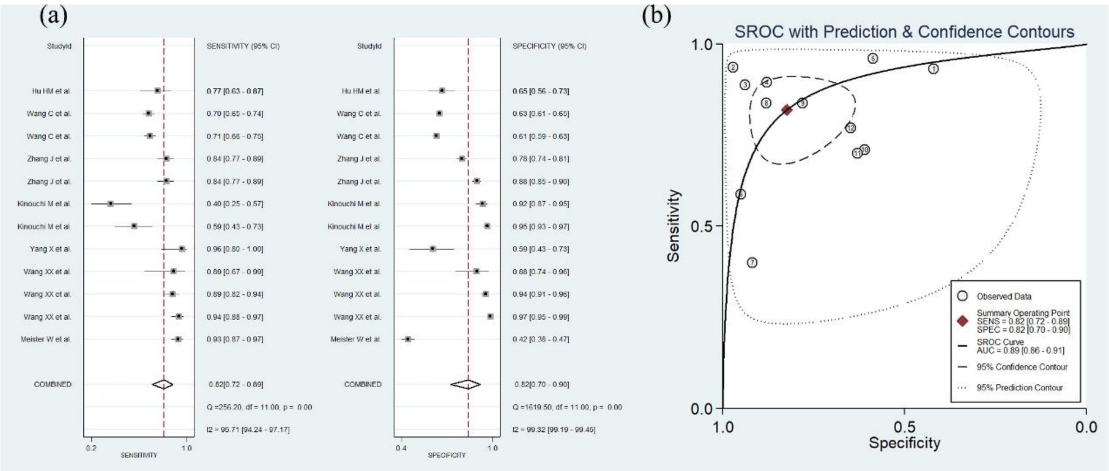

Supplemental Figure 6 Subgroup analysis - Based on the predictor (clinical factors and serological indicators or omics analysis)

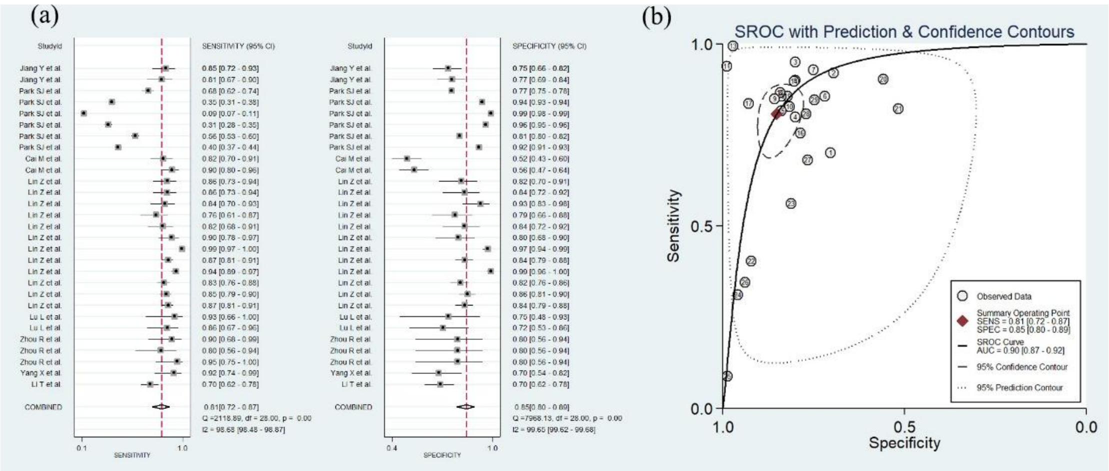

Supplemental Figure 7 Subgroup analysis - Based on the cohort (prospective)

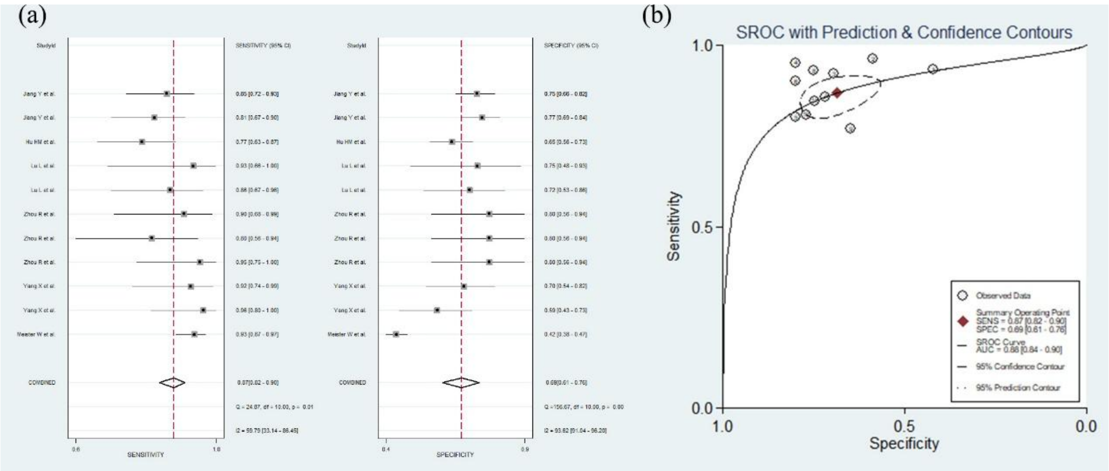

Supplemental Figure 8 Subgroup analysis - Based on the cohort (retrospective)

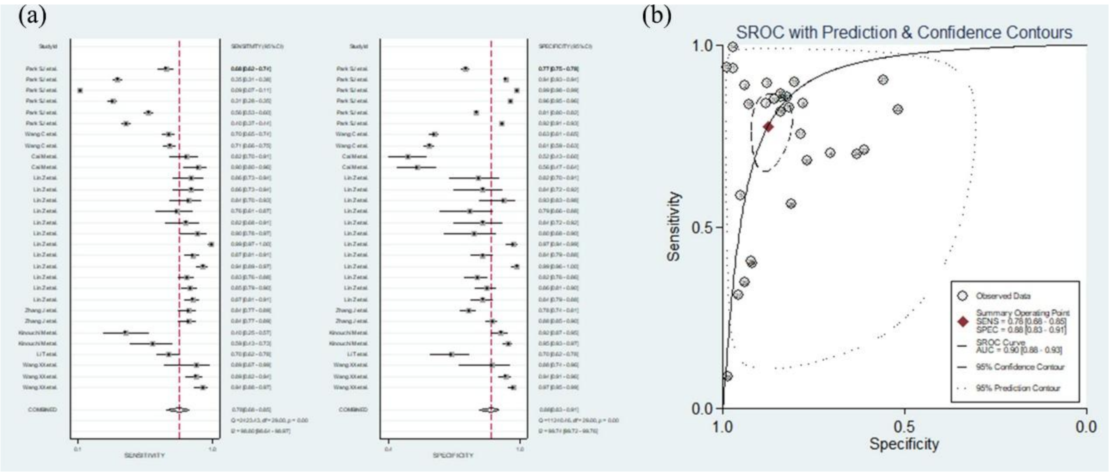

Supplemental Figure 9 Subgroup analysis - Based on the definition of PHN (three months)

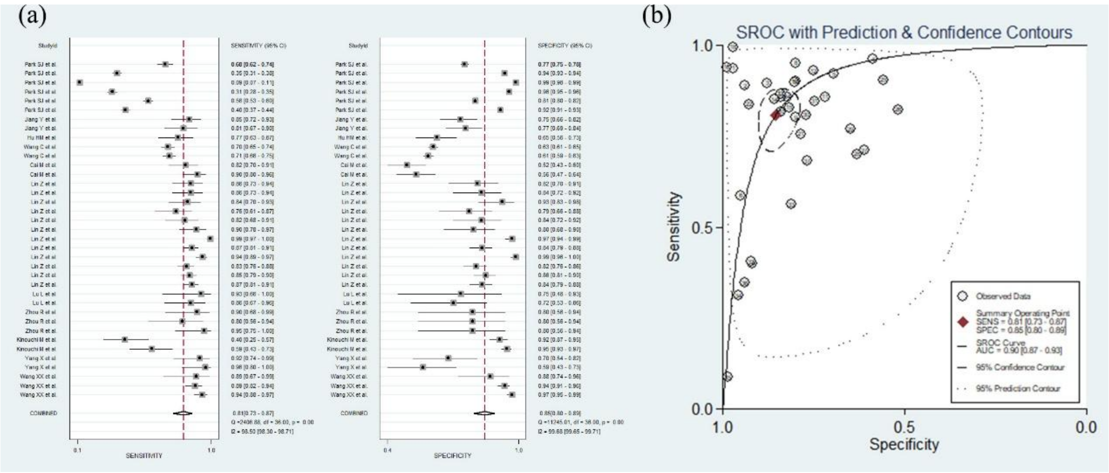

Supplemental Figure 10 Sensitivity analysis

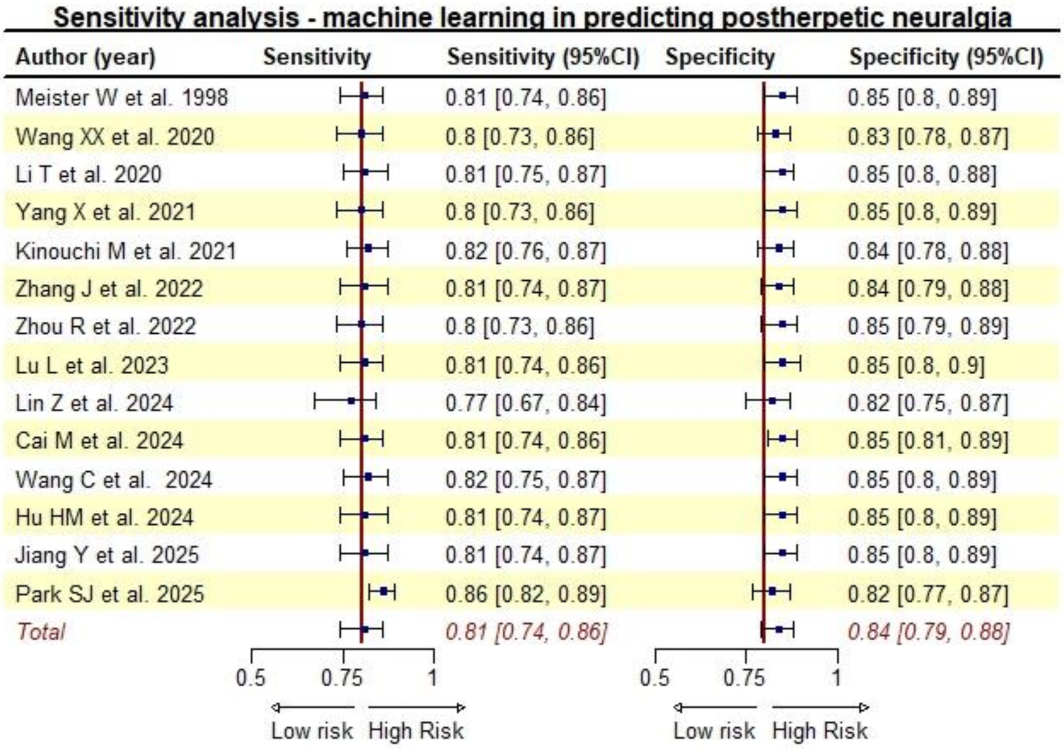

Reference:

1. Meister W, Neiss A, Gross G, Doerr HW, Höbel W, Malin JP, et al. A prognostic score for postherpetic neuralgia in ambulatory patients. Infection. 1998;26(6):359-63.
2. Wang XX, Zhang Y, Fan BF. Predicting Postherpetic Neuralgia in Patients with Herpes Zoster by Machine Learning: A Retrospective Study. Pain and therapy. 2020;9(2):627-35.
3. Li T, Wang J, Xie H, Hao P, Qing C, Zhang Y, et al. Study on the related factors of post-herpetic neuralgia in hospitalized patients with herpes zoster in Sichuan Hospital of Traditional Chinese Medicine based on big data analysis. Dermatologic therapy. 2020;33(6):e14410.
4. Yang X, Shen Y, Ding Z, Tian Y, Hu J, Guo Q, et al. Circulating Level of Myelin Basic Protein Predicts Postherpetic Neuralgia: A Prospective Study. The Clinical journal of pain. 2021;37(6):429-36.
5. Kinouchi M, Igawa S, Ohtsubo S, Doi H, Honma M. Easy-to-use prediction model for postherpetic neuralgia. The Journal of dermatology. 2021;48(10):1622-3.
6. Zhang J, Ding Q, Li XL, Hao YW, Yang Y. Support Vector Machine versus Multiple Logistic Regression for Prediction of Postherpetic Neuralgia in Outpatients with Herpes Zoster. Pain physician. 2022;25(3):E481-e8.
7. Zhou R, Li J, Zhang Y, Xiao H, Zuo Y, Ye L. Characterization of plasma metabolites and proteins in patients with herpetic neuralgia and development of machine learning predictive models based on metabolomic profiling. Frontiers in molecular neuroscience. 2022;15:1009677.
8. Lu L, Mei L, Li X, Lin Y, Wang H, Yang G. Metabolomics profiling in predicting of post-herpetic neuralgia induced by varicella zoster. Scientific reports. 2023;13(1):14940.
9. Lin Z, Yu LY, Pan SY, Cao Y, Lin P. Development of a Prediction Model and Corresponding Scoring Table for Postherpetic Neuralgia Using Six Machine Learning Algorithms: A Retrospective Study. Pain and therapy. 2024;13(4):883-907.
10. Cai M, Yin J, Zeng Y, Liu H, Jin Y. A Prognostic Model Incorporating Relevant Peripheral Blood Inflammation Indicator to Predict Postherpetic Neuralgia in Patients with Acute Herpes Zoster. Journal of pain research. 2024;17:2299-309.
11. Wang C, Song X, Liu J, Song Y, Gao J. Analysis of Risk Factors and Development and Validation of a Dynamic Nomogram for Postherpetic Neuralgia: A Retrospective Study. Journal of pain research. 2024;17:3935-48.
12. Hu HM, Mao P, Liu X, Zhang YJ, Li C, Zhang Y, et al. A Nomogram Model for Predicting Postherpetic Neuralgia in Patients with Herpes Zoster: A Prospective Study. Pain physician. 2024;27(8):E843-e50.
13. Jiang Y, Huang Z, Sun W, Huang J, Xu Y, Liao Y, et al. Roseburia intestinalis-derived butyrate alleviates neuropathic pain. Cell host & microbe. 2025;33(1):104-18.e7.
14. Park SJ, Han J, Choi JB, Min SK, Park J, Choi S. Deciphering risk factors for

severe postherpetic neuralgia in patients with herpes zoster: an interpretable machine learning approach. Regional anesthesia and pain medicine. 2025.
